# Smart Bioinoculants for *Arachis hypogaea*: Controlled Release of *Bradyrhizobium* and the Role of Naringin in Symbiosis Enhancement

**DOI:** 10.3390/plants14111601

**Published:** 2025-05-24

**Authors:** Adriana Belén Cesari, Natalia Soledad Paulucci, Marta Susana Dardanelli

**Affiliations:** 1 Departamento de Biología Molecular, Facultad de Ciencias Exactas, Físico-Químicas y Naturales, Universidad Nacional de Río Cuarto, Ruta Nacional 36, Km 601, Córdoba X5804BYA, Argentina; acesari@exa.unrc.edu.ar (A.B.C.); npaulucci@exa.unrc.edu.ar (N.S.P.); 2 Instituto de Biotecnología Ambiental y Salud (INBIAS), Consejo Nacional de Investigaciones Científicas y Tecnicas, Rio Cuarto X5800BIA, Argentina

**Keywords:** alginate beads, *Bradyrhizobium*, naringin, control release, peanut (*Arachis hypogaea* L.), symbiosis

## Abstract

Peanut (*Arachis hypogaea* L.) is one of the most important oilseeds crops worldwide. Through symbiosis with the bacterium *Bradyrhizobium* sp., peanuts can assimilate atmospheric nitrogen, reducing the need for chemical fertilizers. However, this nitrogen fixation process is highly sensitive to environmental factors that can inhibit the early stages of symbiotic interaction. In this study, we propose the encapsulation of *Bradyrhizobium* sp. SEMIA6144 and the flavonoid naringin (Nar) in alginate beads to improve flavonoid stability and promote nodulation kinetics in peanuts. Three types of beads were synthesized: A (control, SEMIA6144 only); B (SEMIA6144 induced with 10 µM Nar); and C (SEMIA6144 co-entrapped with 1 mM Nar). Although Nar increased cell mortality (2-fold compared to control) and reduced metabolic activity—particularly at 1 mM—cells in beads B and C responded by altering their membrane fatty acid profile (30% and 55.5% of 18:1, respectively) leading to a reduction in saturated fatty acids (5.8% and 13.1% for 16:0 and 18:0 in B; 11.8% and 21.2% in C). Bacterial release kinetics followed a primarily Fickian diffusion model, with minor matrix–bacteria interactions in Nar-treated beads. Notably, bacterial release in peanut root exudates was 6%, 10%, and 11% higher for beads A, B, and C, respectively, compared to release in physiological solutions. Nar-beads enhanced the formation of curved root hairs, promoted bacterial colonization in root hair zones, and stimulated the appearance of rosette-like structures associated with nodule initiation. In conclusion, encapsulating *Bradyrhizobium* sp. SEMIA6144 with Nar in beads represents a promising strategy to improve symbiotic nitrogen fixation in peanuts.

## 1. Introduction

Peanut (*Arachis hypogaea* L.) (is one of the world’s major oil crops, with Argentina being the world’s largest exporter of high-quality peanuts. A limiting factor for peanut growth and productivity is the availability of nitrogen. Its supply can be achieved through biological nitrogen fixation (BNF), an economical and sustainable alternative [1], whose utilization can be optimized using inoculants containing rhizobia, particularly strains of the *Bradyrhizobium* genus. The establishment of BNF symbiosis between legumes and rhizobia is a complex process involving numerous steps and is strongly affected by the microecology of the rhizosphere, such as soil temperature, pH, texture, salinity, deficiency of essential elements, which can inhibit the initial stages of the symbiotic association [2]. In peanuts, rhizobia entry occurs via crack entry, a process that takes place at the junction of a root hair cell and adjacent epidermal cells, where a rosette is formed, or in openings created by the emergence of lateral roots [3]. *Bradyrhizobium* has been shown to induce nodulation in peanuts via the nodulation factors (NF)-dependent mechanism [4]. It was reported that metabolites such as flavonoids present in root exudates (RE) showed a significant role in the interaction with bacteria of the genus *Bradyrhizobium* [5,6,7], attracting rhizobia to the root surface and activating specific genes in rhizobia to produce NF [1,5,8]. Importantly, the nitrogen fixation efficiency of peanuts is relatively low compared to other legume species, allowing for improvements [9]. One potential strategy to improve this process involves the induction of *nod* genes in rhizobia using specific flavonoids; for example, exogenous application of genistein has been shown to accelerate plant-bacteria signaling and mitigate the adverse effects of low root zone temperature on nodulation and nitrogen fixation in soybean [9,10]. The pre-incubation of *Bradyrhizobium japonicum* with genistein has been shown to accelerate the onset of nitrogen fixation, as well as increase nodule number, nodule size, and overall plant growth [11]. Similarly, the isoflavonoid naringenin may enhance nodulation, possibly by inducing the expression of *Rhizobium leguminosarum nod* genes [12].

Naringenin, a potent inducer of the *nod* gene in *R. leguminosarum*, is taken up by a rapid, passive and highly reversible process in which the flavonoid simply dissolves in the lipid matrix [13]. However, one of the majority flavonoids secreted by the peanut is the glycosylated flavonoid naringin (Nar) [14]. Little is known about how the pre-incubation of rhizobia with glycosylated flavonoids affects nitrogen-fixing symbiosis in peanut plants. Some authors showed that flavonoid glycosides degrade rapidly in non-sterile soil (3 to 12 h in non-sterile soil) and do not seem stable, however they can still be responsible for symbiotic effects as they are continuously replenished by living plants [15]. The half-life of flavonoid glycosides can be short due to their rapid hydrolysis by both plant and microbial glycosidase enzymes [16]. This rapid degradation of flavonoids in soil limits their effectiveness in promoting symbiosis between rizhobia and legumes, reducing their potential to enhance nodulation and symbiosis establishment. Given this limitation, encapsulation can provide a solution.

Despite encapsulation of microorganisms for agronomic purposes, these have been studied in recent years [17,18,19,20], these mainly focused on the protection of microorganisms, without addressing the stability and controlled release of signaling molecules such as flavonoids. To date, no studies have been reported that evaluate the encapsulation of flavonoids together with microorganisms for soil application, which represents a knowledge gap in the development of technologies to optimize nodulation in legume crops. In this framework, we propose the encapsulation of *Bradyrhizobium* sp. and the flavonoid naringin in alginate beads as a strategy to improve flavonoid stability and promote nodulation kinetics in peanuts. These systems protect the encapsulating molecules from the external environment and off-target absorption, thereby increasing their biodistribution and half-life time.

Sodium alginate is commonly used for microorganism and metabolite immobilization due to its biocompatibility, biodegradability, non-toxicity, and easy gel-forming ability [21]. In agriculture, alginate has been used to encapsulate, protect and release soil growth-promoting microorganisms [19,21,22]. Polymers can also release drugs through swelling mechanisms with large amounts of water to release an entrapped compound through simultaneous swelling-diffusion mechanisms [23]. In addition to these two processes, the release of the entrapped material may be based on degradation of the polymer, or a conjunction of all three mechanisms: diffusion, swelling and degradation. The thickness and permeability of the microsphere determine the release rate of the compound [23], the most common release mechanism for these systems being Fick diffusion [24,25].

Sustainable agriculture could benefit from the encapsulation of bacteria and flavonoids, as the controlled release of signaling molecules, such as flavonoids or NF, can enhance plant-derived signals, strengthening plant–microbe interactions. This approach has the potential to promote and accelerate BNF while reducing the need for fertilizer application. Based on this premise, our study aimed to analyze the physiological behavior of *Bradyrhizobium* sp. SEMIA6144 immobilized on biodegradable beads, pretreated with naringin, or co-entrapped with it, and evaluated its effect on nodulation kinetics in peanut plants. Although *Bradyrhizobium* infection in peanut occurs via “crack entry” and does not require root hair invasion, we hypothesized that naringin might induce early root morphological responses, such as root hair curling and rosetting, possibly through the stimulation of NF synthesis, thereby promoting bacterial accumulation at key infection sites and increasing the efficiency of the symbiotic process.

## 2. Materials and Methods

### 2.1. Bacterial Strain and Culture Conditions

*Bradyrhizobium* sp. SEMIA6144, provided by MIRCEN/FEPAGRO (Porto Alegre, Brazil), was used in this study. This strain is taxonomically classified in the NCBI database (accessed on 1 February 2025) under the taxonomy ID 312252 (https://www.ncbi.nlm.nih.gov/datasets/taxonomy/312252/), where taxonomic and sequence-related information is available.

The strain was cultured in yeast extract mannitol (YEM) medium [26] until the early exponential phase (96 h). For pre-incubation with Nar, 10 μM was added to the medium at 72 h of bacterial growth, and cultures were incubated until 96 h with shaking at 150 rpm in an orbital shaker (Allied, Fisher Scientific, Waltham, MA, USA). Bacterial cultures (1 × 10^9^ CFU·g^−1^) were used for subsequent entrapment.

### 2.2. Entrapment Conditions of Bradyrhizobium *sp.* SEMIA6144

Three conditions of alginate beads were synthesized using the ionic gelation method [27,28]: **A.** SEMIA6144 control; **B.** SEMIA6144 induced with Nar (Sigma-Aldrich, St Louis, MO, USA); **C.** SEMIA6144 co-entrapped with Nar. For bead A, 25 mL of bacterial culture in the stationary phase was centrifuged at 10,000 rpm for 10 min, and the resulting pellet was resuspended in sterile PS. This was then mixed with 20 mL of a sterile 5% sodium alginate (Sigma-Aldrich) solution. In the synthesis of bead B, 25 mL of Nar-induced bacterial culture, also grown to the stationary phase, was combined with 20 mL of a sterile 5% sodium alginate solution. For bead C, 25 mL of stationary phase bacterial culture was centrifuged at 10,000 rpm for 10 min, and the pellet was resuspended in a 1 mM Nar solution (co-entrapped). This mixture was then combined with 20 mL of a sterile 5% sodium alginate solution.

Once the beads were obtained by dripping into a 2% CaCl_2_ solution, they were separated from the suspension, washed three times with 500 mL of saline solution, and stored in sterile vials at 4 °C for up to 6 months (short-term storage).

To calculate the yield of the bead synthesis process, the weight of the fresh beads produced, and the total weight of all reagents used in the synthesis were considered. The yield in percentage, was calculated using Equation (1) [19,29].

(1) $$Encapsulation\;process\;yield\;\left(\%\right)=100\times \frac{m\left(beads\right)}{m\left(bacteria\;suspension\;added\;to\;the\;synthesis\right)+m\left(Alginate\right)},$$

where *m*: mass.

### 2.3. Viability Characterization of Immobilized Cells

An amount of 1 g of beads from each condition and storage time (T0 and T6) was dissolved in 3 mL of citrate buffer (pH 7) for 30 min at 25 °C. Bacterial counts were performed using the microdroplet technique described by [30] on solid YEM medium, and the plates were incubated at 28 °C for 5 days.

To determine the percentages of live and dead cells within the beads, the LIVE/DEAD^®^ BacLight™ Bacterial Viability Kit (Molecular Probes^®^, Life Technologies™, Waltham, MA, USA) was used in conjunction with a Guava^®^ easyCyte flow cytometer (Cytek^®^ Biosciences, Fremont, CA, USA). The stock dye solutions were prepared as follows: propidium iodide (PI, 30 µM) and SYTO9 (6 µM) from the LIVE/DEAD™ BacLight kit (Invitrogen, Molecular Probes, Carlsbad, CA, USA) were used according to the manufacturer’s instructions. The bacterial biomass obtained from beads A, B, and C was stained with 3 µL of a SYTO9-PI mixture (1:1) and incubated in the dark at room temperature for 15 min prior to analysis [31]. Under blue excitation, intact cells appear green due to staining with SYTO9 and permeabilized cells appear red due to additional or substituted PI intercalation of acids into nucleic acids. Flow cytometer (FC) measurements were performed using an excitation at 488 nm from a blue laser. Optical filters were set to measure red fluorescence at 695/50 nm (RED-B or FL3) and green fluorescence at 525/30 nm (GRN-B or FL1). For instrument calibration, one- and two-color controls were utilized. Unstained SEMIA6144 cells served as background control. Once the populations were localized using double-stained controls (SYTO9: PI mixture) for live and dead cells, bacterial samples extracted from beads A, B, and C stained with the SYTO9: PI mixture were analyzed. Data obtained from the FC were analyzed using FlowJo_V10 software (FlowJo LLC, Ashland, OR, USA).

### 2.4. Determination of the Effect of Naringin on Cellular Respiratory Activity and ROS Production

The effects of Nar on respiratory activity and reactive oxygen species (ROS) production of SEMIA6144 cells, were characterized by FC. A total of 1 g of T0 beads A, B, and C was dissolved in 3 mL of citrate buffer and subsequently centrifuged at 10,000 rpm for 10 min. 5-cyano-2,3-ditolyl tetrazolium chloride (CTC, Sigma-Aldrich, St Louis, MA, USA) was used to evaluate the respiratory activity of cells. For CTC staining, 10 µL of CTC 50 mM was added to 90 µL bacterial suspensions to make up final dye concentrations of 0.5 mM in the samples, and then incubated at 37 °C with shaking (250 rpm) for 30 min. After finishing these staining procedures, samples were washed with PS three times, the concentration of the bacterial suspension was adjusted to about OD600 = 0.1, and the samples were placed on ice for FC analysis using a Guava^®^ easyCyte system (Cytek^®^ Biosciences, Fremont, CA, USA), with excitation by an argon laser at 530–550 nm and emission at 590 nm. Cell free and kill controls were run to check for abiotic reduction to formazan. CTC fluorescence intensity was acquired during FC and the average respiration intensity of the cells was characterized using the average fluorescence intensity [32]. Data obtained from the FC were analyzed using FlowJo_V10 software.

Dichloro-dihydro-fluorescein diacetate (DCFH-DA) is a total ROS probe (Sigma-Aldrich). DCFH-DA is a nonfluorescent, cell membrane-permeable molecule that is hydrolyzed by intracellular esterases to produce dichlorodihydrofluorescein (DCFH), which is then oxidized by ROS, producing the fluorescent compound 2′,7′-dichlorofluorescein [33]. According to the manufacturer’s protocols, 1 mL of the cell suspension extracted from T0 beads A, B, and C were incubated with 3 µL of the probe (10 µM) and then incubated for 30 min at 28 °C shaking at 8250 rpm. After incubation, the cell suspension was washed, centrifuged and the pellet was resuspended in 1 mL of PS. Green fluorescence was assessed using a Guava^®^ easyCyte system (Cytek^®^ Biosciences, Fremont, CA, USA), a 488 nm laser, and a 530/30 nm bandwidth filter. Data are expressed as the percentage of positive cells. Cut-off values were established using an unstained sample. Data obtained from the FC were analyzed using FlowJo_V10 software.

### 2.5. Naringin Intracellular Content

A total of 1 g of T0 and T6 beads B and C were dissolved in 3 mL of citrate buffer, then centrifuged at 10,000 rpm for 10 min. To the pellets, 1 mL of 90% ethanol was added and heated at 80 °C for 60 min. Subsequently, 2 mL of chloroform was added, and the mixture was centrifuged at 8000 rpm for 15 min. The supernatant, containing the released Nar, was used to determine its content by measuring the absorbance at 285 nm using UV-visible spectrophotometer (Beckman DU 640, Brea, CA, USA) and comparing it with a standard curve. Finally, the Nar content of each condition was expressed as µg·mg protein^−1^ [34].

### 2.6. Membrane Fatty Acids Composition of Immobilized Bacteria

Total lipids from bacteria immobilized from T0 and T6 beads A, B, and C, were extracted according to the method of [35]. The lipid phase was methylated using boron trifluoride (20% methanol) and heated at 100 °C for 2 h to obtain fatty acid methyl esters (FAMEs). FAMEs from SEMIA6144 cells were analyzed using a 5890 II gas chromatograph (Hewlett-Packard, Palo Alto, CA, USA) equipped with a highly polar HP 88 column, and a flame ionization detector was employed. The gas chromatograph conditions were described by [19]. FAMEs were identified by comparing their retention times with those of commercial standards (Sigma Chemical Co., St. Louis, MO, USA).

### 2.7. Chemical Beads Characterization

First, the samples were dried for 48 h at 30 °C under a dynamic vacuum system. Subsequently, the chemical composition of the beads was analyzed by FTIR spectroscopy in the attenuated total reflectance (ATR) mode. For this purpose, spectra were obtained for the compounds to be encapsulated and for the components of the bead shell. The FTIR equipment used was a Spectrum Two FTIR spectrometer (PerkinElmer, Shelton, CT, USA), equipped with a MIRacle^®^ ATR accessory (Pike Technologies, Fitchburg, WI, USA). The spectra were obtained with a resolution of 8 cm^−1^ and a data collection of 16 scans.

### 2.8. Bead Swelling Behavior

The swelling degree (*SD*) of the beads was determined according to the method described by [36]. A total of 1 g of dry beads (*W_D_*) was weighed and immersed in 10 mL of sterilized 0.9% NaCl solution for 24 h. Excess water from the swollen beads was removed with filter paper and filtered with water. Excess water from the swollen beads was removed with filter paper and weighed (*W_S_*). The swelling of the alginate beads was evaluated at time intervals of 0, 1, 2, 4, 8, and 24 h by recording the weight of the beads before and after immersion in PS. The *SD*, in percentage, was calculated using Equation (2) [36].

(2) $$SD=\frac{\left({W_{S}-W}_{D}\right)}{W_{D}}\times 100\left(\%\right),$$

where *W_S_* and *W_D_* are the weights of the swollen and dry samples, respectively.

### 2.9. In Vitro Release of Bacteria and Flavonoid from Beads

We placed 1 g of beads A, B, and C in PS (pH 7) and peanut RE solution (pH 5) for 15 days at room temperature (25 °C). To assess the release of bacteria from beads, 1 mL aliquots were taken at predetermined time intervals and replaced with an equal volume of fresh solution. The RE was obtained from a hydroponic system in which 7-day-old peanut seedlings were grown in Hoagland solution [37], as detailed in [14].

To study the release kinetics of entrapped microorganisms, the technique described by [38] modified according to [19] was used. A 100 μL sample of bead suspension was taken to determine the number of colony-forming units using the microdroplet technique. The result was expressed as the cumulative number of cells. Moreover, the amount of Nar in the collected samples was analyzed by Lambda 950 UV–Vis Spectrophotometer (Beckman DU 640, Brea, CA, USA) in the region of 285 nm, against a calibration curve. The results were expressed as the cumulative release (µg·mL^−1^) of Nar. As blank measurements, analogous experiments were performed with solutions containing empty beads. The assays were performed for triplicate.

### 2.10. Release Mechanism Prediction

For a deeper understanding of the release profile of the cumulative viable bacteria from the swellable capsule system, mathematical modeling of the active ingredient release from swellable polymeric systems was applied [39,40,41]. The release kinetics of bacterial and Nar release from the beads was analyzed by applying the semi-empirical model Equation (3) as proposed by Ritger and Peppas [42] as it considers the Fickian diffusion phenomenon and the relaxation phenomenon of polymer chains:

(3) $$\frac{M_{t}}{M_{\infty }}=k\times t^{n},$$

where *M_t_* and *M_∞_* represent the amount of material released at time *t* and at equilibrium, respectively, *k* is the characteristic constant of the compound–polymer system, and *n* is the characteristic diffusion exponent of the release mechanism, which allows us to determine the phenomena present during the release process.

When *n* = 0.5, the release of the compound follows a Fickian-type diffusion mechanism. An anomalous or non-Fickian diffusion occurs when 0.5 > *n* < 1. A quasi-fickian diffusion process takes place at *n* < 0.5, in cases where the matrix is a porous material and, therefore, there is a combined partial diffusion, either through a swollen matrix or water-filled pores. Values of *n* < 0.50 denote the existence of another process simultaneous to diffusion. In the case of *n* = 1, the kinetics of the release system are of zero order. The release process is controlled by the relaxation of the polymer chains with diffusion occurring at a constant rate, only if the geometry of the system does not change during the release process.

### 2.11. Pot Experiment and Nodulation Kinetics Assay

To determine whether inoculation with beads containing the induced microorganism, or the co-presence of Nar, accelerates nodulation events, nodulation kinetics were monitored. The SEMIA6144 *m*Cherry labeled strain (on loan from Dr. Maitrayee DasGupta [43], which fluoresces and allows for the observation of rooting and/or nodular primordia, was previously prepared.

Seeds of *Arachis hypogaea* L. (peanut) cv. Granoleico (proved by El Carmen S.A, General Cabrera, Córdoba, Argentina) were surface-sterilized as described by [44] and germinated at 28 °C in Petri dishes containing 0.8% water agar. Germinated peanut seeds were placed in vermiculite pots and subjected to the following treatments (a) no inoculation; (b) inoculation with 1 g of bead A; (c) inoculation with 1 g of bead B; (d) inoculation with 1 g of bead C. The poles containing the seedlings were placed in a growth chamber at 24 °C, with illumination for 16 h/day [45]. Roots of plants were observed and analyzed at 7, 11, 15, 20, 25, and 33 days post-inoculation using a fluorescence microscope (Nikon Eclipse 50i; Nikon Corporation, Tokyo, Japan). Additionally, the primordia were examined with an Axiophot optical microscope (Carl Zeiss, Buenos Aires, Argentina S.A.). For this purpose, roots were washed with a 5% sodium hypochlorite solution for 20 min and then stained with 1% methylene blue.

### 2.12. Statistical Analysis

All experiments were carried out in triplicate. One-way ANOVA was used to analyze the results. When ANOVA indicated a significant treatment effect, the least significant differences test (Tukey, *p* < 0.05) was applied to compare the mean values. Data were analyzed using InfoStat software, version 2018I (Grupo InfoStat, FCA, Universidad Nacional de Córdoba, Argentina). In addition, a principal component analysis (PCA) was used to visualize the general dispersion patterns between the different types of beads (A, B, and C) and to verify the correlation with the set of parameters measured in bacteria (viability, live and dead cells, ROS) and symbiotic characteristics in relation to the plant (number of rosette appearances).

## 3. Results and Discussion

### 3.1. Viability and Metabolic Bacteria Activity of SEMIA6144 in New Beads

**Figure 1:**
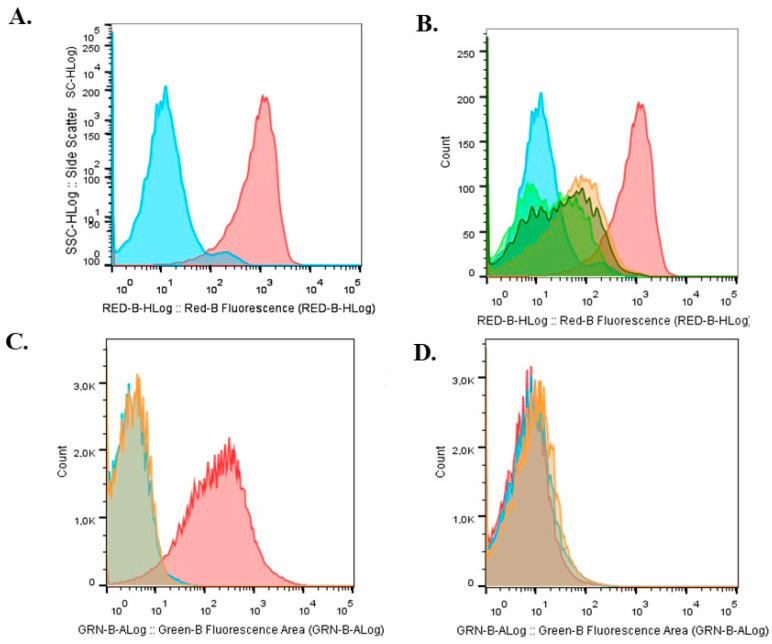
Metabolic activity of bacteria extracted from beads of different conditions stained with CTC (5-cyano-2,3-bis(4-methylphenyl)-2H-tetrazolium). (**A**) Monoparametric histograms obtained from FC analysis of a population of metabolically active (red) and inactive (light blue) cells. (**B**) Histograms of bacteria extracted from bead A (orange), bead B (light green), and bead C (dark green). Monoparametric histograms obtained by FC analysis using the DCFA-DA probe. (**C**) Autofluorescent cells (light blue), Nar-treated cells (orange), and cells treated with hydrogen peroxide and the probe (red). (**D**) Bacteria extracted from bead A (light blue), bead B (red), and bead C (orange). Representative image of three independent experiments.

In this work, the viability and metabolic state of bacteria immobilized in alginate was characterized, both in the absence and presence of Nar. Table 1 shows the synthesis yield percentages for the beads, which ranged between 74% and 79%, with viable bacterial immobilization values of 1.10^8^–1.10^9^ CFU·g^−1^. Using the LIVE/DEAD^®^ bacLight™ bacterial viability kit and flow cytometry, it was observed that 96% to 97% of the cells remained viable on the beads recently synthesized, maintaining the integrity of its membrane, while a small percentage (0.57% to 1.4%) were dead cells in all beads, but more marked in the B and C beads. This suggests the existence of a fraction of the cell population in an intermediate metabolic state that cannot be classified as either alive or dead, which might correspond to cells with partially damaged membranes [31]. Flavonoids, depending on their concentration, can exert damage to the cell envelope such as membrane permeabilization, membrane rupture or an uncontrolled increase in ROS molecules, with consequent oxidative damage and lipid peroxidation events [46,47]. In this study, the result reveals that the presence of Nar in beads B and C (T0) increased cell mortality 2-fold and 2.5-fold, respectively, compared to bead A. (Table 1). To assess whether Nar induced cellular damage, bacterial respiratory activity was evaluated in T0 beads by reduction assay of the CTC probe to an insoluble and fluorescent formazan salt (Figure 1A,B). Bacterial cells with electron transport system activity or respiratory activity can reduce 5-cyano-2,3-ditolyl tetrazolium chloride (CTC) to an insoluble fluorescent CTC-formazan product that accumulates inside the cells [48,49,50]. Figure 1A, shows two regions of CTC formazan relative fluorescence, depending on the fluorescence intensity of positive and negative controls [49,51]. Positive control refers to metabolically active cells and negative control refers to cells inactivated by heat (100 °C). When comparing the different experimental conditions (Figure 1B), variations in bacterial metabolic activity were detected depending on naringin concentration. Bead A showed an intermediate proportion of active cells. Bead B (light green) displayed a bimodal distribution, with a significant fraction of cells shifted toward the inactive population, suggesting that this concentration might partially reduce metabolic activity. In contrast, bead C (dark green) showed a higher percentage of active cells compared to bead B, although with lower fluorescence intensity than bead A, suggesting that this concentration may have a different effect on bacterial viability. These results indicate that 10 µM Nar may negatively affect the metabolic activity of a bacterial subpopulation, whereas 1 mM Nar does not appear to cause as strong an inhibitory effect as observed at 10 µM. Some authors reported inhibitory effects on the activity of some enzymes caused by flavonoids, which may be due to interactions with key enzymes involved in different anabolic and catabolic pathways, such as fatty acid and sterol synthesis, cell wall cross-linking, Krebs cycle, and glucose metabolism [46,52]. To determine whether the application of Nar induces oxidative stress in the cells, we analyzed cells extracted from T0 beads (A, B, and C) using the DCFH-DA probe (Figure 1C,D). It was confirmed that Nar does not fluoresce in the probe’s detection range (Figure 1C). When the probe-treated cells were analyzed, it was found that cells from bead A exhibited a basal oxidative stress level of 14%. In contrast, cells exposed to 10 µM Nar showed an increase in oxidative stress of 19.3%, representing a 5.3% rise. Similarly, cells treated with 1 mM Nar exhibited an oxidative stress level of 20%, corresponding to a 6% increase compared to basal stress levels (Figure 1D).

In summary, using alginate and the ionic gelation method, we were able to efficiently encapsulate an optimal amount of CFU/mL of SEMIA6144 both in the absence and presence of Nar. The presence of flavonoids induced a slight oxidative stress that could be responsible for the lower metabolic activity of the bacteria in beads A and B. Nevertheless, the bacteria obtained from the three types of beads show optimal viability and metabolic activity to be considered in biological activity assays in interaction with peanut plants.

### 3.2. Viability of SEMIA6144 in 6-Month Beads

**Figure 2:**
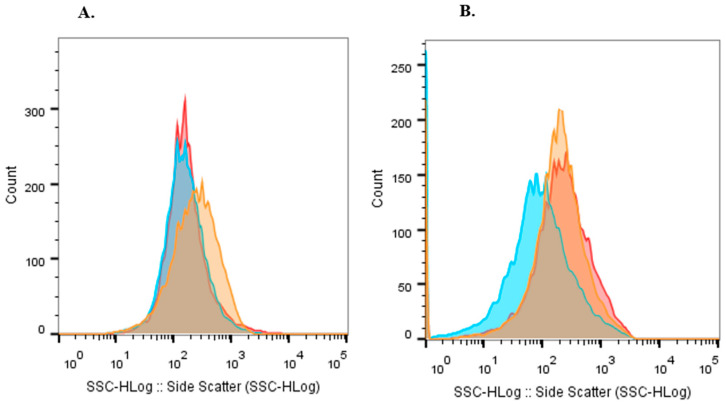
Population complexity analyzed by FC with live and dead cells staining from beads of (**A**). Storage time: 0 months and (**B**). Storage time: 6 months. Light blue: bead A; Red: bead B; Orange: bead C.

For the effective application of alginate-based inoculants, the encapsulated cells must remain viable both during storage and after soil introduction, ensuring their proper release from the beads [19,22,53]. In this study, we store the beads at 4 °C for 6 months (T6). A decrease in the viable cell count was observed compared to freshly prepared beads (Table 1). The percentage of viable cells was 16.25%, 13.6%, and 5% for condition A, B, and C, respectively. However, the number of viable cells inside the beads (7.8·10^7^, 1.5·10^8^, 8.6·10^7^ CFU·g^−1^) is still an optimal value for use as an inoculum [54]. This long-term survival of encapsulated cells indicates that these beads may have sufficient protective capacity for applications in biofertilizers [55]. LIVE/DEAD^®^ bacLight™ bacterial staining revealed that the T6 beads still contained a high percentage of live cells (96–97%), while the percentage of dead cells increased compared to the T0 of storage (Table 1). Also, Figure 2 shows that the population’s complexity increased with storage time, and Nar increased the complexity of the population stored for 6 months at 4 °C. Increased complexity may indicate the presence of additional subpopulations, including fully viable cells (intact membranes), dead cells (compromised membranes), and cells in a transient state with partial membrane permeability, potentially experiencing stress or an intermediate metabolic condition. This heterogeneity could result from a differential cellular response to treatment, where not all cells react uniformly.

The cell membrane is one of the main target sites for flavonoids against microorganisms. These compounds are in the hydrophobic fractions of the lipid bilayer of the membrane and cause a change in its fluidity and rigidity [56]. Nar can preferentially localize in the polar lipid bilayer of the membrane and exert a sorting effect in the hydrophobic region of this compartment [57]. In this study, we quantified the intracellular uptake of Nar in SEMIA6144 cells immobilized under conditions B and C (Table 1). At storage T0, the intracellular incorporation of Nar was 0.4 µg naringin·µg protein^−1^ in condition B, while in condition C, it reached 0.7 µg naringin·µg^−1^ protein. After T6 storage, intracellular naringin levels remained stable in both conditions at 0.6 µg naringin·µg^−1^ protein [13], which showed that naringenin can be accumulated inside the cell of *Rhizobium* spp., reaching a cellular concentration up to 80 times higher than the extracellular concentration and that no evidence of intracellular metabolism of the compound was recorded. Although not described for the glycosylated flavonoid Nar, some authors reported that naringenin and luteolin accumulation is not saturable at concentrations up to at least 600 nM, is independent of incubation time, and that decreasing the temperature to 4 °C will result in a substantial increase in accumulation [13,34].

### 3.3. Physicochemical Characterization of Beads

**Figure 3:**
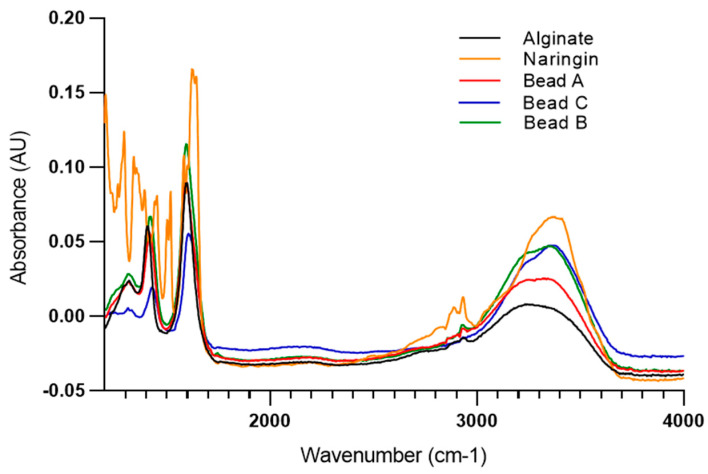
ATR spectra of alginate, naringin and beads A, B, and C.

**Figure 4:**
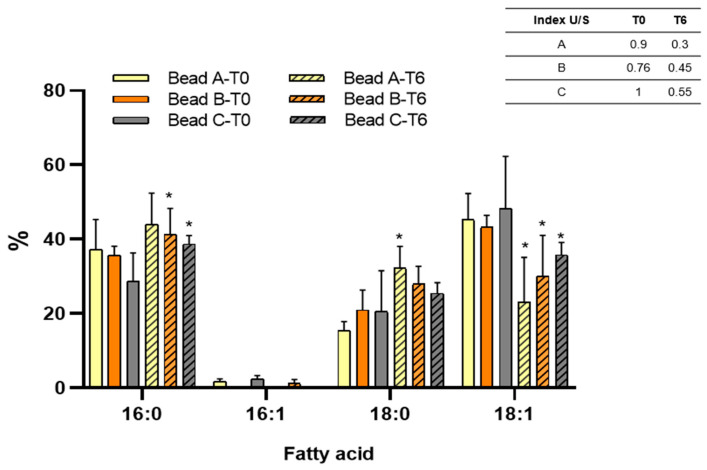
Fatty acid composition of *Bradyrhizobium* sp. SEMIA6144 extracted from bead A, B, and C after storage at 4 °C. Asterisks indicate statistically significant differences in each fatty acid compared to freshly prepared beads (0 years of storage). Values represent the mean ± SEM of three independent experiments. Statistical significance was determined using a two-way ANOVA followed by Tukey’s post hoc test (* *p* < 0.05).

ATR spectroscopy was used to characterize the beads under different conditions and to confirm the presence of Nar (Figure 3). The alginate spectrum displayed prominent peaks in the range of 1000–1200 cm^−1^, corresponding to the vibrations of C–O and C–C groups in the polysaccharide backbone. The strong and broad absorption band characteristic of O–H stretching in sodium alginate powder was consistent with findings from previous studies [58,59]. In the spectrum of Nar, the intense O–H stretching absorption observed from 3600 to 2600 cm^−1^ indicates the presence of a carboxylic group in the compound. The strong and broad hydrogen bonded O–H stretching bands centering 3300 and 3400 cm^−1^ are for alcohols and phenols [60]. The absorption peaks observed between 1456 and 1238 cm^−1^ can be attributed to groups containing double bonds. Characteristic bands of Nar were identified at 1645 cm^−1^, corresponding to the vibrations of C=C bonds in the aromatic ring, at 1519 cm^−1^ for aromatic groups, and at 1363 cm^−1^ for phenolic O–H groups, consistent with standard spectra of Nar reported in the literature [61]. In previous work, we characterized alginate beads containing immobilized *Bradyrhizobium* and *Azospirillum* [19]. Figure 4 illustrates that in bead A, an increase in the peak within the 3400–2700 cm^−1^ region is observed, which corresponds to O–H stretching vibrations and suggests enhanced hydration of the bacterial cells (bound water). In bead B, the peak around 3400 cm^−1^, associated with O–H groups, is more pronounced compared to bead A. In bead C, the presence of Nar is more evident, as indicated by the higher intensity of the peaks at 3400 cm^−1^ (O–H) and 1600 cm^−1^ (aromatic) compared to bead A and bead B, confirming the presence of Nar.

### 3.4. Membrane Fatty Acids Composition of Immobilized Bacteria

The changes in fatty acid composition regarding chain length, isomer profile, and the presence of alkyl side chains are an essential adaptative mechanism of bacteria to tolerate situations that cause damage or disruption of the membrane [62]. Flavonoids, due to their amphipathic nature, can be inserted into the lipid bilayer of bacterial membranes. This interaction may alter fatty acid composition, membrane fluidity and permeability, compromise its integrity, and impact cellular viability. Figure 4 presents the fatty acid profile of the SEMIA6144 entrapped under different conditions. In freshly prepared beads (T0), bacteria extracted from bead B and C showed a reduction of 4.3% and 23% in 16:0 fatty acid content, respectively, along with a 35% increase in 18:0 fatty acid content, compared to cells from bead A. Despite this fatty acid remodeling, the unsaturated-to-saturated (U/S) ratio remained largely unaffected.

After six months of storage at 4 °C, an increase in 16:0 and 18:0 saturated fatty acids, along with a decrease in 18:1 unsaturated fatty acid, was observed in all beads, leading to a lower U/S ratio in entrapped bacteria compared to those extracted from fresh beads. This storage effect was previously reported by our group for *Bradyrhizobium* sp. SEMIA6144 stored in beads A for up to four years [22]. However, compared to bacteria from bead A, those extracted from bead B showed a reduction of 5.8% and 13.1% in 16:0 and 18:0 saturated fatty acids, respectively. Similarly, bacteria from bead C exhibited an 11.8% and 21.2% reduction in 16:0 and 18:0 saturated fatty acids, respectively, compared to A. On the other hand, an increase of 30% and 55.5% in 18:1 unsaturated fatty acid content was observed in bacteria extracted from B and C, respectively, compared to bead A. These results indicate that the presence of the naringin causes a remodeling of the bacterial membrane, in particular by decreasing saturated fatty acids and increasing unsaturated fatty acids. Similarly, previous studies have reported that the exposure of *E. coli* cells to naringenin resulted in a significant increase in long-chain unsaturated fatty acids, accompanied by a reduction in cyclopropane and saturated fatty acids, with these effects becoming more pronounced as the naringenin concentration increased from 0 to 2.20 mM [63]. Comparable modifications in membrane fatty acid composition have also been observed in Gram-negative bacteria, *Escherichia coli* and *Salmonella typhimurium*, upon exposure to phenolic compounds derived from plant extracts [64,65]. Consistent with our findings, *Staphilococcus xylosus*, *Staphilococcus carnosus*, and *Micrococcus luteus*, naringenin and biflavonoids predominantly caused a reduction in specific fatty acids while slightly increasing the relative proportion of longer-chain fatty acids [66].

The membrane modifications suggest that they are part of a detoxification mechanism or can diminish the effects of membrane disturbance caused by flavonoids. These changes, particularly the increase in longer-chain fatty acids, suggest an enhancement of the hydrophobic membrane barrier in microorganisms [66].

### 3.5. Bead Swelling and Release of Bacterial and Naringin

**Figure 5:**
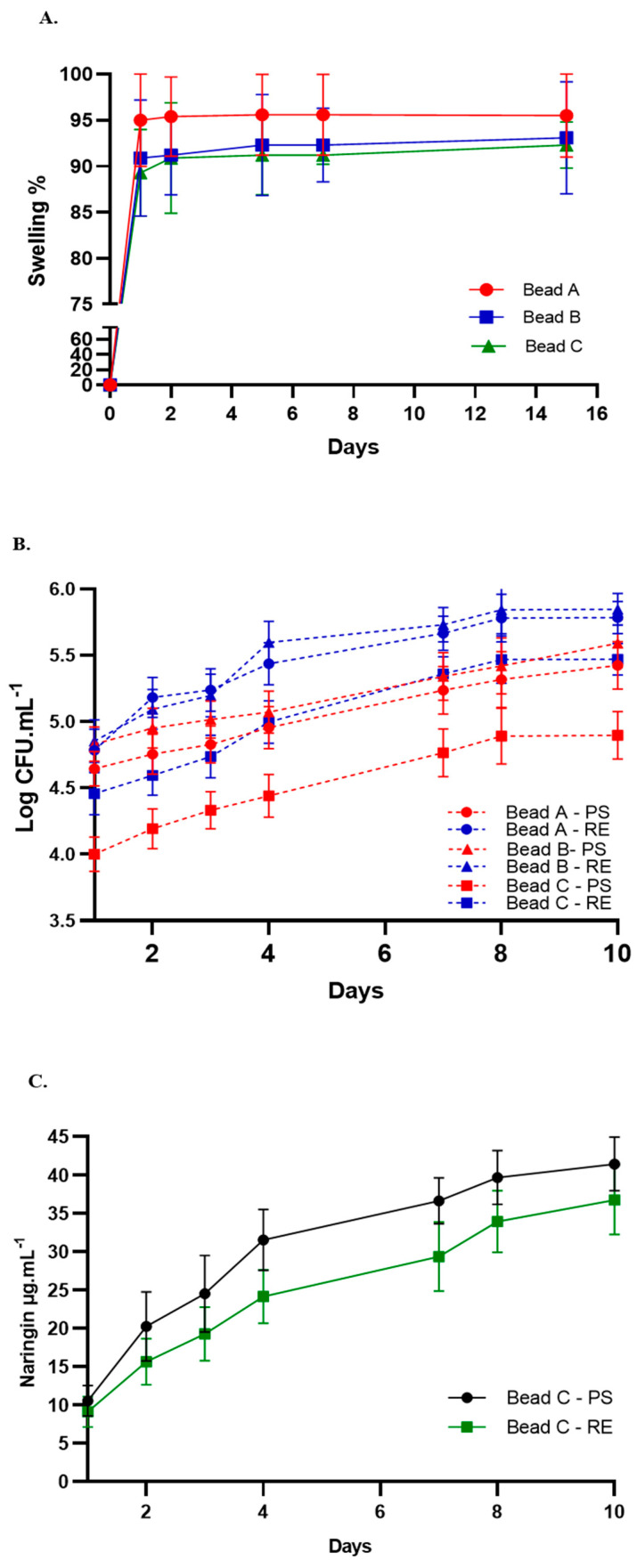
(**A**). Swelling ratio of alginate beads A, B, and C conditions in 0.9 wt % NaCl solution vs. time (days). The data present mean ± standard deviation calculated from at least three technical replicates. (**B**). Release behavior of microorganisms from alginate beads immersed in PS (red line) and in peanut RE (blue line); (**C**). Release of naringin from Cbeads immersed in PS or 7-day peanut RE.

Swelling and moisture content are critical parameters for assessing water absorption and release in microcapsules, directly influencing the release of bacterial or molecules into the soil [59]. Figure 5A illustrates the swelling index for all formulations, which ranged between 92% and 95%. Equilibrium swelling of the beads was achieved within 1 day, and the swelling remained nearly constant for up to 15 days in solution. Beads from condition A reached a swelling percentage of 95% at 24 h, maintaining this value through day 15. In contrast, B and C exhibited a swelling percentage of 90% at 24 h, which increased to 93% and 92%, respectively, by day 15 (Figure 5A). The presence of naringin affected the swelling behavior of the beads, with bead A exhibiting the highest swelling degree (Figure 5A). Similarly, other authors have reported that for alginate–starch–bentonite beads encapsulating *Routella planticola*, equilibrium swelling was reached after 1 day and remained almost constant for up to 8 days in solution [55]. Additionally, for alginate beads encapsulating *Pseudomonas fluorescens*, a swelling index of 78% was achieved within 24 h [59].

The release kinetics confirmed that bacteria, regardless of pre-treatment, were capable of being released from the beads into the external environment. In PS, bead A and B demonstrated the highest bacterial release over the 10-day study period (Figure 5B). In peanut RE, the kinetics of bacterial release were higher for all bead types compared to those in PS (Figure 5B). After 15 days, the number of bacteria released in peanut RE was 6%, 10%, and 11% higher for A, B, and C, respectively, compared to their release in PS. The initial phase of bacterial release (0–1 days) was short and rapid, likely due to the swift release of bacteria from the outer layer and surface of the capsules, coinciding with the rapid swelling of the capsules upon water absorption. Between days 1 and 15, the release process became more gradual, as the swelling of the capsules approached equilibrium within the medium. During this phase, the release rate slowed, while the cumulative release continued to increase steadily, indicating that bacterial release dominated this stage. This behavior has been previously described by other authors, including the release of *R. planticola* Rs-2 from alginate–starch–bentonite beads [55] and the release of *Pseudomonas fluorescens* from alginate–whey protein concentrate beads [59]. RE are typically composed of primary metabolites such as sugars, amino acids, and carboxylic acids, along with a wide variety of secondary metabolites. In addition to serving as carbon and nitrogen substrates for microbial growth, RE compounds exert multiple effects on rhizosphere microbes by acting as signaling molecules, attractants, and stimulants [67]. The composition of the peanut radical exudates was previously described [14] and was found to be mainly composed of flavonoids, auxins tryptophan, organic acids, fatty acids, and terpenes. The presence of these molecules in the medium could explain the higher release of bacteria under these conditions. These results are highly significant, as these molecules are expected to play a crucial role in the peanut rhizosphere by triggering the controlled release of bacteria from the beads in sufficient numbers.

Also, we have reported that RE contains 0.05 μg·L^−1^ of citric acid as one of its components [14]. This organic acid has a high capacity to chelate Ca^2+^ ions, as demonstrated by [68]. The chelation of Ca^2+^ by these compounds could weaken the cross-linking of alginate, thus facilitating the release of bacteria immobilized in the polymeric matrix, which could justify the increased bacterial release in the presence of RE.

Additionally, the release kinetics of naringin from bead C were evaluated, showing that this molecule can diffuse from the bead into the surrounding environment, both in PS and in peanut RE (Figure 5C). However, the release was higher in PS (Figure 5C). The porous structure of low alginate permits the easy diffusion of encapsulated compounds to an external solution [69]; however, since RE contains a variety of flavonoids, a plausible hypothesis could be competition for diffusion channels or interaction with the alginate matrix between Nar and other structurally similar flavonoids. This competition could reduce the amount of Nar released into the surrounding environment. However, further studies are needed to confirm these specific mechanisms.

### 3.6. Release Mechanism Studies

As shown in Table 2, the release profiles obtained from the different bead formulations were best described by the Ritger–Peppas model, a semi-empirical approach that accounts for both Fickian and non-Fickian diffusion mechanisms in polymer-based delivery systems [70,71]. This model includes two constants: *k*, which reflects the structural and geometrical features of the polymer matrix and the encapsulated compound, and *n*, the release exponent that characterizes the type of release mechanism.

When studying the bacteria release mechanism in PS, the ‘n’ values were 0.12 and 0.11 for bead A and B, respectively, which indicates that both beads present a predominantly Fickian release, while bead C presented an ‘n’ of 0.18, this value indicates that the release is Fickian, but with some matrix–bacteria interaction. Regarding the release in RE, ‘n’ values between 0.06 and 0.1 were recorded, which represents an almost completely Fickian release. When the release of the flavonoid naringin was analyzed, in PS and RE, the values of ‘n’ were 0.45 and 0.53, respectively, indicating that the release in RE is more influenced by the relaxation of the matrix, possibly due to the interaction with components of the medium.

The *k* values calculated for beads A, B, and C in both PS and RE indicated that the release of bacteria is slower in the beads containing Nar. This means that there is a stronger interaction between Nar and alginate. Also ‘*k*’ reflects the initial release rate, higher values of ‘*k*’ indicate faster release. In Table 2, the values of ‘*k’* are higher in PS than in RE, for beads A and B, indicating that the release is faster than in rhizodeposition. The presence of naringin at a concentration of 1 mM reduces the ‘*k*’ value, indicating a delayed release effect. Fickian diffusion refers to a release process controlled exclusively by diffusion, based on Fick’s laws. This type of diffusion describes how molecules or particles (in this case, bacteria or Nar) move from a region of high concentration (inside the bead) to a region of low concentration (external medium) due to a concentration gradient. Fickian diffusion is a release mechanism that has been widely associated with polysaccharide-based devices used for the encapsulation of agrochemicals and drugs [24,72].

The release system studied provides a feasible method for the controlled application of biofertilizers across a wide range of climate and soil conditions.

### 3.7. Bacterial Colonization of Peanut Root and Nodulation Kinetic

**Figure 6:**
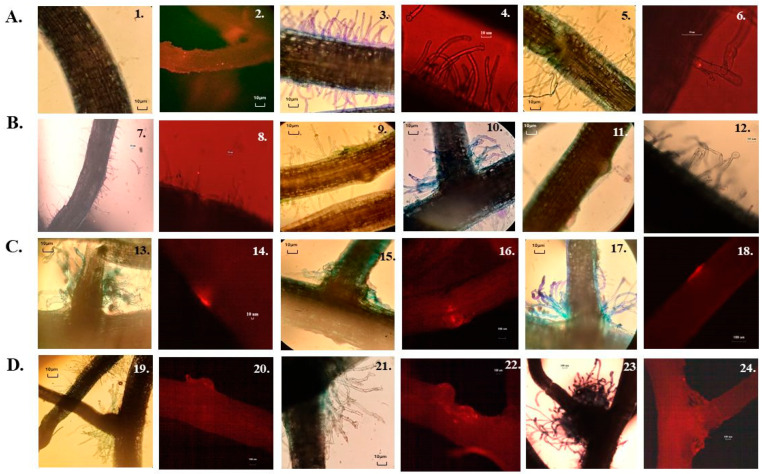
Evaluation of the nodulation kinetics of peanut (*Arachis hypogaea* L.) roots inoculated with beads immobilizing *Bradyrhizobium* sp. SEMIA6144 mCherry, under the three study conditions, by observation of roots stained with methylene blue observed in a brightfield. In addition, by fluorescence microscopy, it was possible to follow nodulation. Panel (**A**): 7 dpi. Bead A: 1–2; Bead B: 3–4; Bead C: 5–6. Panel (**B**): 11 dpi. Bead A: 7–8; Bead B: 9–10; Bead C: 11–12. Panel (**C**): 15 dpi. Bead A: 13–14; Bead B: 15–16; Bead C: 17–18. Panel (**D**): 21 dpi. Bead A: 19–20; Bead B: 21–22; Bead C: 23–24.

The colonization of lateral root bases and root hairs of *Arachis hypogaea* by immobilized fluorescent *Bradyrhizobium* sp. SEMIA6144 Cherry was analyzed using fluorescence microscopy. Brightfield microscopy of methylene blue-stained roots provided structural details, while fluorescence microscopy enabled the identification of colonization zones and the formation of nodule primordia (Figure 6). Regardless of the type of pearl, SEMIA6144 colonized the lateral roots of peanut inoculated plants, forming annular fluorescent rings, indicating consistent root colonization. Interestingly, in *Arachis*, which is typically described as nodulating independently of NF, symbiosis was always associated with the curling or branching of rosette-like root hairs on lateral roots. The perception of bacterial NF typically results in both temporal and spatial distinct morphological, physiological, and molecular responses in roots [9]. Our results showed that at 7 days post-inoculation (dpi) (Figure 6A), plants inoculated with bead A exhibited sparse root hair development. However, fluorescence microscopy confirmed bacterial colonization, as indicated by the presence of red fluorescence clusters on the root surface (2). In contrast, plants inoculated with beads containing *Bradyrhizobium* sp. SEMIA6144 pre-induced with 10 µM Nar (bead B) or co-entrapped with 1 mM Nar (bead C) showed enhanced root hair formation and increased bacterial colonization in the root hair zone (3, 5) compared to control beads. Notably, plants inoculated with bead B displayed curved root hairs (4), a known response to NFs in legumes [73]. Similar effects have been reported in *Phaseolus vulgaris* inoculated with *Rhizobium etli* pre-induced with naringenin (1.5 μM) [74], where exposure to naringenin-induced cultures resulted in an increased number of relatively short, curved root hairs.). This structural adaptation could facilitate bacterial accumulation near root hairs and crack entry points, enhancing infection efficiency. Root hair curvature might create a microenvironment that improves *Bradyrhizobium* adhesion and accumulation, increasing the likelihood of successful symbiosis establishment.

By 11 dpi (Figure 6B), plants inoculated with bead A exhibited increased root hair development on lateral roots (7, 8), accompanied by bacterial colonization. In contrast, plants inoculated with bead B and C showed the emergence of rosette-like structures at lateral root junctions (10), pronounced root hair curling (12), and early nodular primordia formation (9, 11). In this context, [43] report that NF is required for the intracellular infection of the basal cortical cell that forms the nodule primordium. By 15 dpi (Figure 6C), plants inoculated with bead B exhibited increased bacterial colonization in rosette zones, with root hairs orienting toward the crack entry zone, where nodular primordia were also observed (16). Similarly, plants inoculated with bead C displayed rosettes and developed nodular primordia (18).

By 21 dpi (Figure 6D), all inoculated plants exhibited rosettes and nodular primordia, with bead B and C treatments leading to macroscopically visible nodular structures (22, 24).

Our findings demonstrate that alginate beads, particularly bead B, effectively promote the delivery of NF from naringin-induced bacterial cultures in a concentrated form to the root system. This, in turn, facilitates morphogenic responses, including biofilm formation, rosette development, and nodular primordia initiation, reinforcing the role of flavonoid signaling in peanut–rhizobia interactions.

### 3.8. Multivariate Statistical Analysis of Principal Components

**Figure 7:**
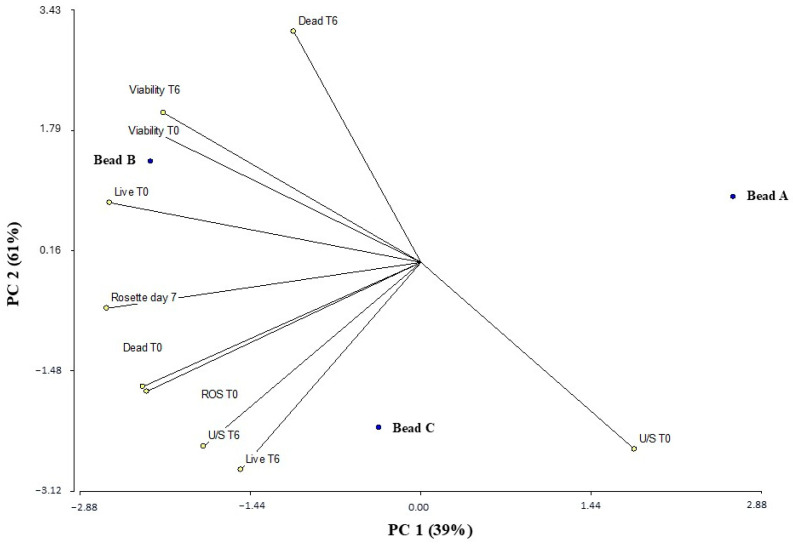
Principal component analysis plot generated by InfoStat software program V. 1.0 (see “Multivariate Statistical Analysis” section) based on biochemical parameters of *Bradyrhizobium* sp. SEMIA6144 cells extracted from beads A, B, and C, at initial time (T0) and after six months of storage at 4 °C (T6). Straight lines represent the correlation vectors of physiological and biochemical variables measured in *Bradyrhizobium* sp. SEMIA6144 cells: viability (T0 and T6), dead cells (T0 and T6), reactive oxygen species (ROS, T0), rosette formation (day 7), live cells (T0 and T6), and unsaturated/saturated fatty acid ratio (U/S, T0 and T6). Isolated points represent the multivariate profiles of bacteria extracted from beads A, B, and C, both at initial time (T0) and after six months of storage at 4 °C (T6). PC1 and PC2 refer to principal components 1 and 2, which explain 39% and 61% of the total variance, respectively.

Principal component analysis (PCA) showed that the first two components (PC1 and PC2) together explained 100% of the total variance in the data, accounting for 61% and 39%, respectively (Figure 7). This indicates that the biplot adequately represents the multivariate structure of the system, and clearly differentiating the profiles of the analyzed samples PCA was performed to explore the multivariate relationship between the variables measured in the samples corresponding to beads A, B, and C. The PCA showed that the first two components explained 100% of the total variance in the data (PC1: 61%; PC2: 39%), justifying the use of the biplot for interpreting the results.

In the biplot, the samples are distributed differently in the PC1–PC2 plane, revealing contrasting physiological and biochemical profiles between the beads. Bead A is projected toward the right end of the PC1 axis, primarily associated with high values of the U/S variable at time 0 (U/S T0). Bead B is in the left quadrant, showing a strong association with the variables viability T0, viability T6, and, to a lesser extent, with dead T6, suggesting a more stable cell condition and greater viability over time.

Meanwhile, bead C is projected at the bottom of the graph, correlated with ROS T0, dead T0, and live T6, indicating a profile associated with greater oxidative stress and mortality at the beginning of the experiment. The orientation and length of the vectors suggest that variables such as dead T6 and viability T6 were particularly influential in sample separation, as were U/S T0 and ROS T0.

Furthermore, it was observed that the appearance of rosettes on day 7 (D7) was primarily associated with bead B, based on its proximity in the multivariate space. This morphological condition, absent from the other beads, could represent a specific cellular reorganization in that condition, possibly related to the sustained levels of viability observed.

## 4. Conclusions

This study demonstrates that it is possible to immobilize large numbers of *Bradyrhizobium* sp. SEMIA6144 cells and naringin in alginate beads. Despite Nar increasing cell mortality and decreasing the metabolic activity of the cells, particularly at higher Nar concentrations (1 mM), SEMIA6144 cells responded to growth with Nar or its co-presence by modifying the membrane fatty acid composition, favoring the accumulation of saturated fatty acids and reducing unsaturated fatty acids.

Our results demonstrate the effectiveness of alginate beads as a vehicle for a gradual release of the bacteria, with higher release rates in PS compared to peanut RE. However, the presence of Nar in the beads also affected their release behavior, with a slower release observed in the presence of 1 mM of Nar, suggesting a retarding effect of naringin on bacterial release. Release kinetics analysis indicated that bacterial release followed a primarily Fickian diffusion model, with slight interaction between the matrix and bacteria in the naringin-treated beads. This type of release could be useful for the controlled application of biofertilizers, allowing for sustained bacterial release.

For the first time, we demonstrate the use of Nar (10 µM and 1 mM) in *Bradyrhizobium* sp. SEMIA6144 immobilized in alginate beads promoted the formation of curved *A. hypogaea* root hairs and bacterial colonization in the root hair zones, favored the emergence of rosette-like structures associated with nodule formation, facilitating bacterial colonization and the development of nodular primordia in the roots.

In summary, these results support the idea of the promoter effect of the flavonoid naringin, improving bacterial colonization and promoting symbiosis in *A. hypogaea*, which could have important implications for the development of more effective microbial inoculants. The results of this research allow us to propose future perspectives, including the nodulation gene induction capacity of the glycosylated flavonoid naringin in *B*. sp SEMIA6144, as well as applications in field trials.

## Figures and Tables

**Table 1:** Beads T0 and T6 months storage at 4 °C. Performance of the bead synthesis process, intracellular incorporation of naringin. Viability (CFU·g^−1^) and percentages of live and dead cells determined by FC.

Condition	Yield (%)	T0				T6			
		Nar (µg·µg^−1^)	Viability (CFU·mL^−1^)	Live %	Dead %	Nar (µg·µg^−1^)	Viability (CFU/L)	Live %	Dead %
Bead A	79.7 ± 5	0	4.8 × 10^8^ a	96 ± 3 a	0.57 ± 0.1 a	0	7.8 × 10^7^ a	95.7 ± 3 a	2.1 ± 0.8 a
Bead B	76.1 ± 4	0.4 ± 0.02 a	1.1 × 10^9^ a	96.6 ± 3 a	1.08 ± 0.2 b	0.6 ± 0.1 a	1.5 × 10^8^ a	96 ± 1 a	3.1 ± 0.9 b
Bead C	74.8 ± 6	0.7 ± 0.2 b	1.8 × 10^9^ a	97 ± 2.8 a	1.41 ± 0.2 c	0.6 ± 0.2 a	8.6 × 10^7^ a	97 ± 2 a	1.09 ± 0.3 b

Different letters indicate significant differences between treatments within each column and for each growth condition, according to one-way ANOVA followed by Tukey’s HSD test *(p* < 0.05).

**Table 2:** Release kinetics parameters of different formulations at PS and RE.

Model Parameters			
**Bacterial release in PS**	**n**	**k**	**r**
Bead A	0.12	0.74	0.95
Bead B	0.11	0.74	0.94
Bead C	0.18	0.64	0.97
**Bacterial release in RE**	**n**	**k**	**r**
Bead A	0.07	0.85	0.95
Bead B	0.06	0.86	0.94
Bead C	0.10	0.78	0.96
**Naringin release**	**n**	**k**	**r**
PS	0.45	0.37	0.97
RE	0.53	0.3	0.99

## Data Availability

Data will be made available upon request.
